# Macrophage imbalance (M1 vs. M2) and upregulation of mast cells in wall of ruptured human cerebral aneurysms: preliminary results

**DOI:** 10.1186/1742-2094-9-222

**Published:** 2012-09-21

**Authors:** David Hasan, Nohra Chalouhi, Pascal Jabbour, Tomoki Hashimoto

**Affiliations:** 1 Department of Neurosurgery, Carver College of Medicine, University of Iowa, Iowa City, IA, USA; 2 Department of Neurosurgery, Thomas Jefferson University and Jefferson Hospital for Neuroscience, Philadelphia, PA, USA; 3 Department of Anesthesia and Perioperative Care, University of California San Francisco, San Francisco, CA, USA; 4 Department of Neurosurgery, University of Iowa Hospitals and Clinics, 200 Hawkins Drive, JCP 1616, Iowa City, IA, 52242, USA

**Keywords:** Aneurysm, Inflammation, Macrophages, M1, M2, Mast cells

## Abstract

**Background:**

M1 and M2 cells are two major subsets of human macrophages that exert opposite effects on the inflammatory response. This study aims to investigate the role of macrophage M1/M2 imbalance and mast cells in the progression of human cerebral aneurysms to rupture.

**Methods:**

Ten patients with cerebral aneurysms (five ruptured and five unruptured) underwent microsurgical clipping. During the procedure, a segment of the aneurysm dome was resected and immunostained with monoclonal antibodies for M1 cells (anti-HLA DR), M2 cells (anti-CD 163), and mast cells (anti-tryptase clone AA). A segment of the superficial temporal artery (STA) was also removed and immunostained with monoclonal antibodies for M1, M2, and mast cells.

**Results:**

All ten aneurysm tissues stained positive for M1, M2, and mast cells. M1 and M2 cells were present in equal proportions in unruptured aneurysms. This contrasted with a marked predominance of M1 over M2 cells in ruptured aneurysms (*p* = 0.045). Mast cells were also prominently upregulated in ruptured aneurysms (*p* = 0.001). Few M1 and M2 cells were present in STA samples.

**Conclusions:**

M1/M2 macrophages and mast cells are found in human cerebral aneurysms; however, M1 and mast cell expression seems to markedly increase in ruptured aneurysms. These findings suggest that macrophage M1/M2 imbalance and upregulation of mast cells may have a role in the progression of cerebral aneurysms to rupture.

## Background

Monocytes originate from bone marrow-derived progenitor cells and do not proliferate in the blood [1-4]. Mononuclear phagocytes develop into morphologically and functionally distinct cell types in response to the tissue microenvironment (e.g., lung alveolar macrophages, Kupffer cells, decidual macrophages) [5,6]. Two major subsets of human macrophages can be defined in atherosclerotic plaques: CD14^high^CD16^low^ macrophages, which typically represent 85% to 95% of monocytes, and CD14^low^CD16^high^ macrophages, which account for the remaining 5 to 15% [2-4,7]. These macrophages play opposite roles in inflammation [2-4,7]. A growing body of evidence suggests that a polarized macrophage population can contribute to systemic and neuroinflammatory diseases [8,9].

Macrophages play a critical role in cerebral aneurysm formation and rupture [10-13]. Macrophage depletion and knockout of monocyte chemotactic protein-1 gene in mice reduce the incidence cerebral aneurysm formation [13]. Recent studies also have revealed that mast cells contribute to various vascular diseases through degranulation and release of cytokines including cerebral aneurysm formation [14,15].

We hypothesize that macrophage imbalance and upregulation of mast cells are more pronounced in ruptured compared to unruptured human cerebral aneurysms. The goal of this study is to provide immunohistological evidence of this hypothesis.

## Material and methods

**Figure 1:**
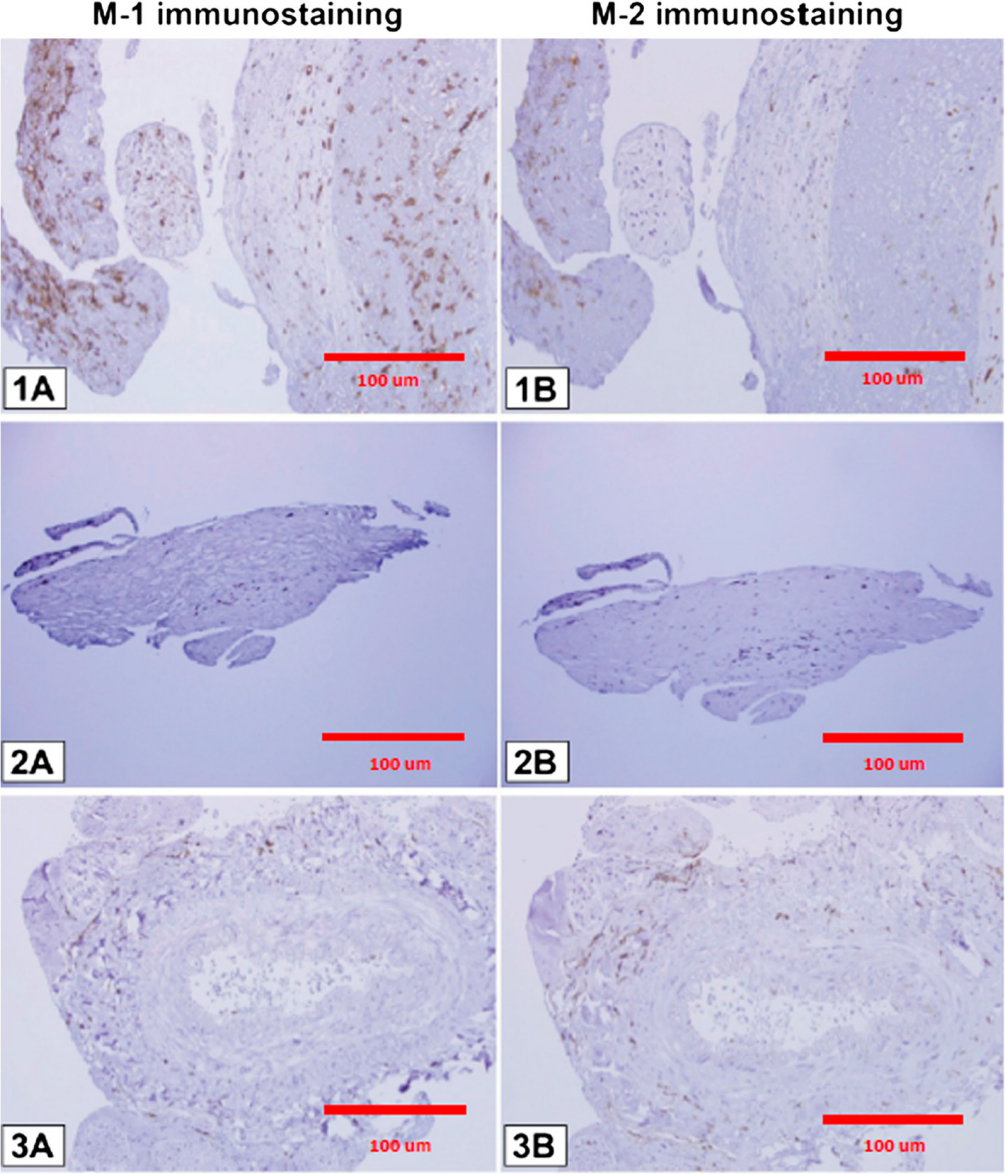
**Immunostaining of monoclonal antibodies with anti-HLA DR for M1 and anti-CD 163 for M2 cells.** In ruptured aneurysms, there is a clear predominance of M1 over M2 cells (1**A**-1**B**). In unruptured aneurysms, M1 cell count is almost the same as M2 (2**A**-2**B**). The expression of M1 and M2 cells is almost similar in STA specimen, but significantly lower than in ruptured and unruptured aneurysms (3**A**-3**B**).

The study was approved by the University of Iowa Institutional Review Board (IRB). Ten consecutive patients with cerebral aneurysms who underwent microsurgical clipping during a 6-month interval were identified. No patients were excluded, except those who were treated with endovascular means. Five patients with unruptured aneurysms and five patients with ruptured aneurysms were included in the study. Mean age was 55 years (range 44–67 years) (Figure 1). Informed consent was obtained in all patients prior to microsurgical clipping. A segment of the aneurysm dome (≥1 mm) and the superficial temporal artery (STA) (≥2 mm) was removed from each patient and placed in formalin. All 20 specimens (10 aneurysms and 10 STAs) were immunostained with monoclonal antibodies to mast cells using anti-mast cells tryptase clone AA1 (DakoCytomation, Carpentaria, CA), M1 cells using anti-HLA DR (ABCAM, Cambridge MA), and M2 cells using anti-CD 163 (ABCAM, Cambridge MA).

**Table 1:** Patient and aneurysm characteristics

**Patient no.**	**Age**	**Sex**	**Aneurysm location**	**Aneurysm size (mm)**	**Rupture**	**M1**	**M2**	**Mast**
					**(SAH)**	**A/STA**	**A/STA**	**cells**
1	50	F	L-ICA	9 × 6	No	1/0	1/0	1
						(13/4)	(11/5)	(4)
2	56	M	R-MCA	5 × 4	No	1/0	1/0	1
						(15/7)	(11/4)	(7)
3	52	F	L-MCA	10 × 10	No	2/0	1/0	1
						(27/3)	(18/6)	(5)
4	67	F	R-MCA	7 × 8	No	2/0	2/0	1
						(26/5)	(27/4)	(3)
5	47	M	R-Pcomm	14 × 11	No	2/0	2/0	1
						(28/7)	(27/5)	(5)
6	68	M	L-MCA	12 × 9	Yes	3/0	1/0	3
						(39/6)	(12/3)	(41)
7	44	M	R-MCA	6 × 5	Yes	3/1	2/1	3
						(41/2)	(22/4)	(39)
8	38	F	A-comm	8 × 8	Yes	3/0	1/0	3
						(37/3)	(14/7)	(31)
9	55	F	L-MCA	9 × 10	Yes	3/0	1/0	3
						(44/6)	(13/4)	(49)
10	74	M	R-PICA	14 × 15	Yes	3/0	1/0	3
						(38/4)	(15/6)	(34)

F = female; M = male; L = left; R = right; ICA = internal carotid artery; MCA = middle cerebral artery; A-Comm = anterior communicating artery; Pcomm = posterior communicating artery; PICA = posterior communicating artery; A/STA = aneurysm/superficial temporal artery; grade 0 = 0–10 cells per HPF, grade 1 = 10–20 cells per HPF, grade 2 = 20–30 cells per HPF, and grade 3 ≥ 30 cells per HPF. Numbers in parentheses are absolute cell counts.

The entire tissue sample collected from the aneurysm dome was embedded in paraffin and analyzed. The sample size, however, differed from one patient to another based on the size of the aneurysm and ease of tissue collection following successful clipping. All fields were analyzed per sample and, for consistency, we chose the field with the highest concentration of cells for semiquantitative analysis. Each field covered the entire section of the aneurysm tissue and included all layers of the wall of the aneurysm dome.

Semiquantitative analysis of the slides was performed based on cell count (immunostained positive cells) per high-power field (HPF) (40×): grade 0 = 0–10 cells per HPF, grade 1 = 10–20 cells per HPF, grade 2 = 20–30 cells per HPF, and grade 3 ≥ 30 cells per HPF. All specimens were assessed by an observer who was blinded to the clinical data and source of tissue.

Statistical analysis was performed using the Kruskal-Wallis test, a nonparametric ANOVA test. This test is used for comparing more than two samples that are independent, or not related. The parametric equivalence of the Kruskal-Wallis test is the one-way analysis of variance (ANOVA). The factual null hypothesis is that the populations from which the samples originate have the same median. When the Kruskal-Wallis test leads to significant results, then at least one of the samples is different from the other samples. The test does not identify where the differences occur or how many differences actually occur.

## Results

**Figure 2:**
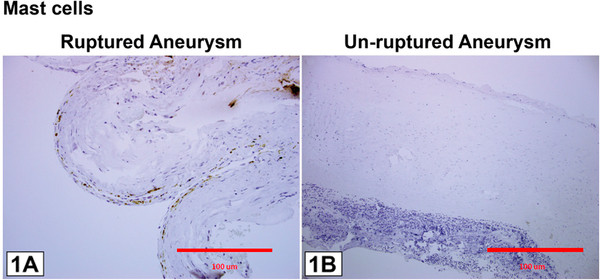
**Immunostaining of monoclonal antibodies for anti-mast cells tryptase (clone AA1).** There are more mast cells visible in ruptured aneurysms than in unruptured aneurysms.

Ten patients with ten aneurysms were included in this study. All ten aneurysms stained positive for expression of M1, M2, and mast cells using anti-HLA DR, anti-CD 163, and anti-mast cells tryptase clone AA1 monoclonal antibodies (Figures 1 and 2).

### Macrophages

Unruptured aneurysms stained almost equally for M1 and M2 cells. Ruptured cerebral aneurysms, however, stained more abundantly with anti-HLA DR monoclonal antibodies, indicating a predominance of M1 over M2 cells. There was only scant staining of M1 and M2 cells in the ten samples of STA tissue (Figure 1).

### Mast cells

Unruptured aneurysms had few mast cells. Ruptured aneurysms had prominently more mast cells stained with monoclonal antibody for anti-mast cells tryptase clone AA1 compared with unruptured aneurysms. There was abundant mast cell staining in STA tissue but this was limited to adventitia and did not extend to the other layers, namely the media, intima, and subendothelium (Figure 2).

### Statistical analysis

**Figure 3:**
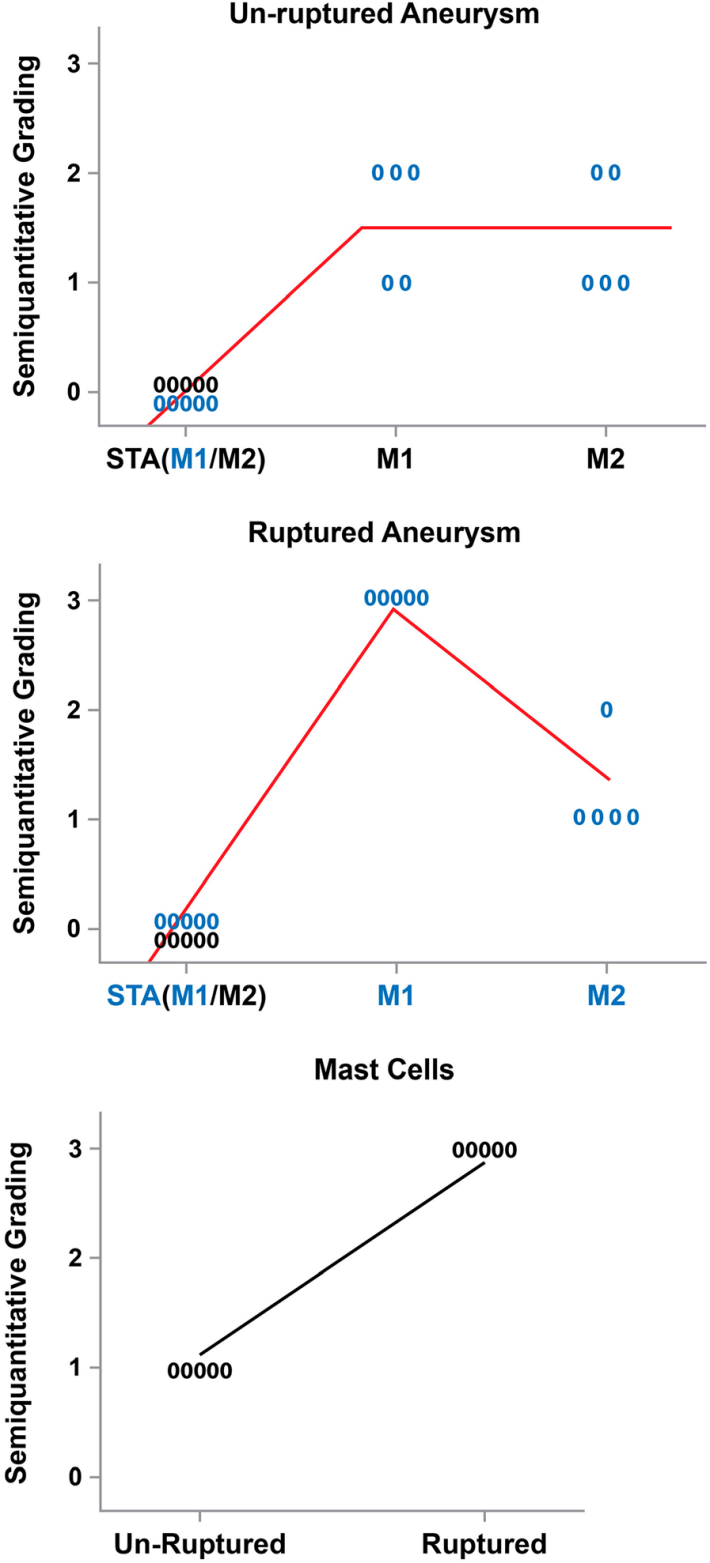
**Semiquantitative grading for M1, M2, and mast cells in ruptured and unruptured aneurysms.** The number of M1 and M2 cells in unruptured aneurysms is almost equal, with significantly more M1 cells noted in ruptured aneurysms. Clearly more mast cells are present in ruptured aneurysms compared to unruptured aneurysms.

Areas immunostained for M1 and M2 cells with cell count differences were localized, and semiquantitative analysis was performed (Figure 3). M1 cell expression was significantly higher than M2 cell expression (*p* = 0.045) in ruptured aneurysms. M1 cell expression was also greater (*p* = 0.001) in ruptured and unruptured aneurysms vs. STA. Mast cell expression was significantly higher in ruptured aneurysms vs. unruptured aneurysms (*p* = 0.001).

## Discussion

### Monocyte differentiation into M1 and M2 subsets

Monocytes differentiate into heterogeneous cells [1-4,16]. Differential expression of selected surface molecules is used to identify monocyte subsets in humans and mice. In humans, there are two distinct subsets of macrophages based on the expression of CD16 [7]. CD14^high^/CD16^low^ cells are the primary subtype of macrophages as they represent 80% to 90% of circulating monocytes and express high levels of the chemokine receptor CCR2 and low levels of CX3CR1. They are poor producers of inflammatory cytokines but release interleukin (IL) 1, IL 13, IL 14, IL 4, IL-10, transforming growth factor-β (TGF-β), and vitamin D. Conversely, CD14^low^/ CD16^high^ monocytes have a CX3CR1^high^/CCR2^low^ phenotype and account for inflammatory mediator production, including tumor necrosis factor-α (TNF-*α*), IL 1, IL 6, IL 12, IL 23, matrix metalloproteinases (MMP), prostaglandins, and leukotrienes [1-4,7]. In mice, monocytes differentiate into two major types of macrophages (M1 and M2) [1-4,6,17,18]. M1 and M2 macrophages play opposite roles during inflammation, although both are present in atherosclerotic lesions. M1 macrophages are differentiated from Ly6C^high^ monocytes. They promote inflammation by producing high levels of IL-2, IL-23, IL-6, IL-1, and TNF-*α*. M2 macrophages, on the other hand, are differentiated from Ly6C^low^ monocytes. They differentiate in the presence of IL-4, IL-13, IL-1, or vitamin D3, tend to produce a large amount of IL-10, and express scavenger receptors, mannose receptors, and arginase [1-5]. Unlike M1 macrophages, these cells promote resolution of inflammation.

Recently, there has been a great deal of interest in macrophage heterogeneity in atherosclerotic lesions, particularly regarding the roles of M1 versus M2 macrophages. There is also evidence that an imbalance in the ratio of M1 to M2 macrophages in advanced atherosclerosis impairs resolution of inflammation *in vitro *[1-5].

### Macrophages and cerebral aneurysms

Macrophage-depleted and monocyte chemotactic protein-1 knockout mice have a reduced incidence of cerebral aneurysms [13]. Macrophages were observed in the wall of hypertension-induced cerebral aneurysms in rats and were reported to secrete extracellular matrix-degrading proteolytic enzymes and induce apoptosis of smooth muscle cells [12]. Additionally, macrophages induce fibrosis through secretion of TGF-β and promote the release of reactive oxygen species, TNF-*α* and IL-1 [10]. Macrophages are also an important source of MMP 2 and 9, which presumably contribute to the reduction of mechanical strength and rupture of aneurysms. MMP-2 is expressed in most cerebral aneurysms, whereas MMP-9 is expressed primarily in aneurysms with atherosclerotic changes [19,20]. Macrophages are an especially important source of growth factors and cytokines that stimulate fibrosis [3].

Platelet-derived growth factor, basic fibroblast growth factor (bFGF), and transforming growth factors alpha (TGF-*α*) and beta are expressed by immunohistochemistry in intracranial aneurysms [20-23]. Transcription of procollagens I and III is promoted by TGF-β, secreted in large part by macrophages [24]. In aneurysms, the progression of smooth muscle cells from a contractile to a synthetic phenotype and the increase in relative numbers of smooth muscle cells (by increased proliferation of smooth muscle cells and myointimal hyperplasia) may compensate for the increased MMP expression and sustain aneurysm wall strength [25]. In concordance with this theory, the aneurysm wall shows, by in situ hybridization, increased synthesis of collagen type III but a slight diminution in expression of collagen type III in immunostaining, probably indicating faster collagen turnover. Mural cells die during aneurysm progression and the extracellular matrix-synthesizing capability is progressively lost, leading to a decrease in tensile wall strength and an increase in susceptibility of the aneurysm to rupture [25].

Based on the classification of atherosclerosis by histological changes, nearly all cerebral aneurysms can be considered atherosclerotic [26]. Features of advanced atherosclerosis (including a core of atheromatous debris, a fibrous cap with macrophages and T-cells) are observed in approximately half of intracranial aneurysms [11,26], and myointimal hyperplasia occurs in the other half [25]. Thrombosis occurs in some cerebral aneurysms, which further amplifies the ongoing inflammatory reaction and wall degeneration, with loss of tensile strength and ultimately aneurysm rupture [24,27-32].

### Mast cells and cerebral aneurysms

Ishibashi et al. [14] examined the role of mast cells in the formation of cerebral aneurysms in an experimental rat model. They found that the number of mast cells was significantly increased in aneurysm walls. They also showed that using mast cell degranulation inhibitors attenuated the chronic inflammatory process in the aneurysm wall. This was evident from the decreased nuclear factor-kappa B activation, macrophage infiltration, and expression of monocyte chemoattractant protein-1, MMP, and interleukin-1beta. They also demonstrated that the degranulation of mast cells led to increased expression and activation of MMP-2 and -9 and induced nitric oxide synthase in cultured smooth muscle cells from rat intracranial arteries. Their results suggest that mast cells play a critical role in aneurysm formation in rats through the induction of inflammation. However, they did not address the question of whether mast cells contribute to the progression of cerebral aneurysms to rupture and whether these cells are present in human cerebral aneurysm tissue.

### Interpretation of current findings

**Figure 4:**
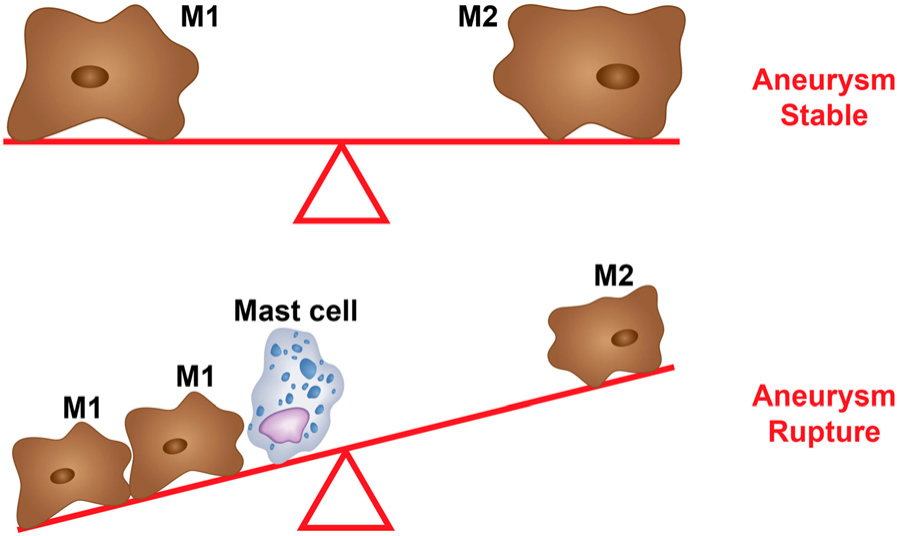
**M1 vs. M2 balance.** Schematic diagram illustrating our hypothesis that a balance between M1 and M2 cells leads to stable aneurysm and that imbalance between M1 and M2 cells with increased population of M1 cells and upregulation of mast cells predisposes the aneurysm to rupture.

**Figure 5:**
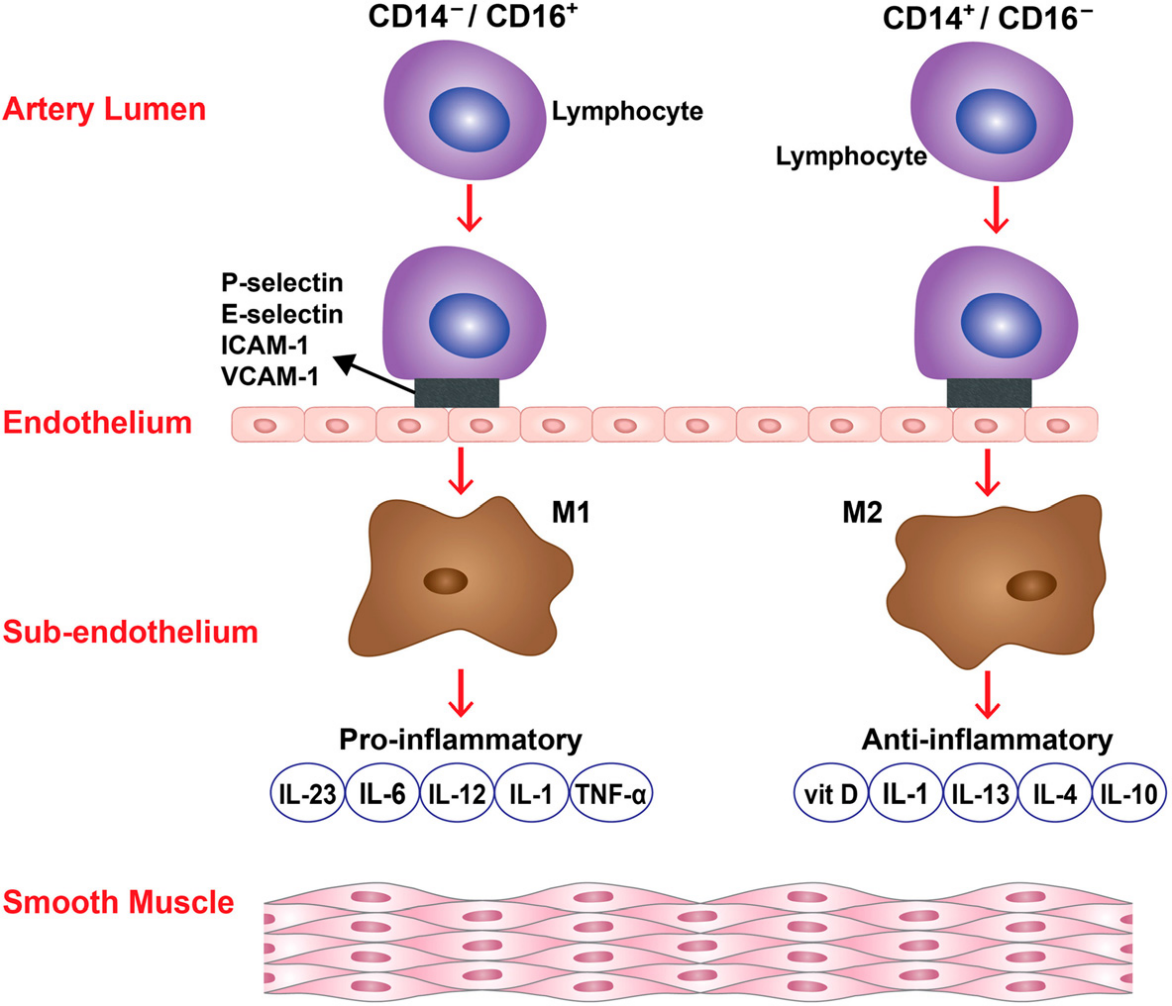
**Polarized monocytes and cytokine production.** Schematic diagram demonstrating two populations of monocytes (CD14^high^ CD16^low^, CD14low CD16^high^) and their polarization to two subtypes of macrophages (M1 and M2) with their cell-specific cytokine production.

**Figure 6:**
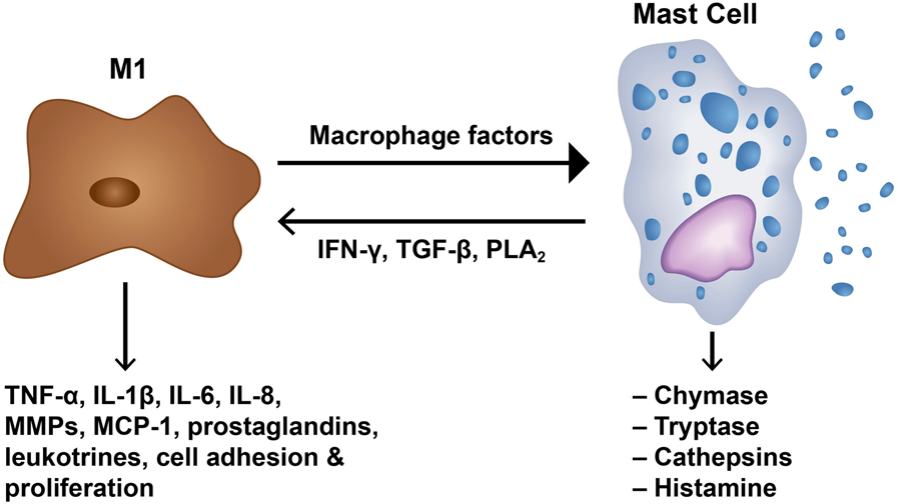
**Mast cell – macrophage interaction.** Schematic diagram demonstrating the interaction of mast cells with macrophages (M1 cells) and their cytokine production implicated in aneurysm formation and rupture.

We found that M1 and M2 cells were present in equal proportions in unruptured aneurysms with very few mast cells, which suggests that the pro- and antiinflammatory activities of M1 and M2 cells, respectively, may be well balanced in the walls of unruptured aneurysms (Figure 4). However, ruptured aneurysms appear to lose this critical M1/M2 balance, as M1 cell expression was significantly more pronounced than M2 cell expression in ruptured aneurysm walls. Additionally, mast cells were found to be significantly upregulated in ruptured cerebral aneurysm tissue. Collectively, these data suggest that a polarized proinflammatory response involving M1 macrophages and mast cells may have a role in the cascade of events leading to aneurysm rupture (Figures 5 and 6). However, the relationship between macrophage subsets and mast cells remains to be defined.

### Limitations

This study is limited by the small sample size, and generalization of the results may not be appropriate. It also is difficult to determine whether increased expression of M1 vs. M2 and upregulation of mast cells in ruptured cerebral aneurysms (compared to non-ruptured) is due to inflammation that occurs following the rupture of the aneurysm or whether there was an increase in expression of these molecules that preceded and led to rupture of the aneurysm.

## Conclusion

M1, M2, and mast cells are expressed in the walls of human cerebral aneurysms. However, M1 and mast cell expression may increase significantly in ruptured aneurysms. Our findings suggest that macrophage M1/M2 imbalance and upregulation of mast cells may play a role in the progression of cerebral aneurysms to rupture. Mast cell activation and M1/M2 macrophage phenotypic modulation may represent important targets for future therapy.

## Abbreviations

STA: Superficial temporal artery; HPF: High-power field; IL: Interleukin; TGF-β: Transforming growth factor-β; TNF-*α*: Tumor necrosis factor-α; MMP: Matrix metalloproteinases; TGF-*α*: Transforming growth factor alpha.

## Competing interests

The authors declare that they have no competing interest.

## Authors’ contributions

DH performed all surgical procedures; conceived and designed the study; acquired, analyzed, and interpreted the data; and drafted the manuscript. NC helped in data analysis and interpretation and helped drafting the manuscript. PJ and TH helped design the study, analyzed and interpreted the data. All authors have revised the manuscript critically for important intellectual content and have given final approval of the version to be published.

## Sources of funding

This study was supported by NIH grant no. R03NS07922 to DH.
